# Optical coherence tomography (OCT) with 2 nm axial resolution using a compact laser plasma soft X-ray source

**DOI:** 10.1038/s41598-018-26909-0

**Published:** 2018-05-31

**Authors:** Przemysław Wachulak, Andrzej Bartnik, Henryk Fiedorowicz

**Affiliations:** Institute of Optoelectronics, Military University of Technology, 2 Urbanowicza Str., 00-908 Warsaw, Poland

## Abstract

We present optical coherence tomography (OCT) with 2 nm axial resolution using broadband soft X-ray radiation (SXR) from a compact laser plasma light source. The laser plasma was formed by the interaction of nanosecond laser pulses with a gaseous target in a double stream gas puff target approach. The source was optimized for efficient SXR emission from the krypton/helium gas puff target in the 2 to 5 nm spectral range, encompassing the entire “water-window” spectral range from 2.3 nm to 4.4 nm wavelength. The coherence parameters of the SXR radiation allowed for the OCT measurements of a bulk multilayer structure with 10 nm period and 40% bottom layer thickness to period ratio, with an axial resolution of about 2 nm and detect multilayer interfaces up to a depth of about 100 nm. The experimental data are in agreement with OCT simulations performed on ideal multilayer structure. In the paper, detailed information about the source, its optimization, the optical system, OCT measurements and the results are presented and discussed.

## Introduction

Optical coherence tomography (OCT) allows one to image an internal structure of the investigated sample in a noninvasive and nondestructive way^1^. Typically, to image a sample, such as multilayer optics, a cross-section using an electron beam is made and the cut is then inspected using an SEM microscope. This, however, damages the sample, or at least small part of it, which for some specific applications, such as testing and inspection of multilayer optics for the EUV lithography, it poses a significant problem.

To overcome this limitation, noninvasive probing techniques, such as OCT were developed^2^. The OCT is performed by splitting an illumination beam into two, namely, a reference beam and an object beam. The object beam is scattered from the sample being investigated, while the reference beam, superimposed with the object beam produces an interference pattern. Due to a broad bandwidth of the illumination and, in turn, small coherence length, the interference occurs only if the delay between the two beams is within a coherence time, related to the coherence length. Thus, it allows one to probe the internal structure of the sample in the direction of the optical axis by changing the delay between the beams (in a time-domain OCT) or by recording the interference in the spectral domain (frequency-domain OCT). Such type of imaging turns out to be a very successful approach to image objects, which cannot be imaged with comparable precision, resolution and in a noninvasive way otherwise. One such object is an eye, and due to its advantages the OCT nowadays became a standard technique to *in vivo* imaging of the internal structure of an eye, such as a retina^3^, to early detect any pathological changes^4^.

The main challenge of visible and near-infrared OCT is the difficulty of observing opaque materials and the axial resolution of typically sub-micron to a few microns. In the OCT the axial resolution depends on the coherence length expressed by Equation (1):

1 $${l}_{c}=\frac{{\lambda }^{2}}{{\rm{\Delta }}\lambda }=\lambda \cdot IRB,$$

where *λ* is the illumination wavelength and *Δλ* is the source bandwidth (i.e. FWHM spectral emission) and *IRB* is an inverse relative bandwidth. For typical broadband near-infrared source of *λ* = 1 μm and *Δλ* = 400 nm (IRB = 2.5) the *l*_*c*_ = 2.5 μm, which for many applications is not sufficient. Moreover, many materials exhibit very low transmission in the VIS and near IR ranges, so it is difficult to scatter the electromagnetic wave out of refractive index discontinuities in the structure of the investigated samples.

As can be seen from Equation (1), even though the IRB has a relatively small value, the central illumination wavelength λ is quite large. Thus, a straightforward way to improve the OCT axial resolution is decreasing the central wavelength to perform so-called X-ray coherence tomography (XCT)^5^ with extreme ultraviolet (EUV) and soft X-ray (SXR) radiation from synchrotrons, high order harmonics generation (HHG) sources and laser-produced plasmas^6,7^. In the EUV/SXR range two possible transmission windows for XCT has been identified: a silicon window (λ = 12–40 nm), dedicated for material science applications, and a well-known “water-window”, dedicated for biology (λ = 2.3–4.4 nm). So far, XCT allowed one to obtain axial resolution of 18 nm in the EUV range, down to 8 nm in the “water-window” range employing a synchrotron source^8^, and 24 nm resolution using an HHG source^9^. XCT in the “water-window” allows resolving in-depth structures of the order of a few nanometers. Moreover, the “water-window” radiation can be used for nanoimaging of biological samples, which exhibit a high natural contrast in this wavelength range due to differences in absorption between carbon and oxygen, present in the biological samples^10^, which allowed for 2-D nanoimaging with resolution better than 15 nm with synchrotrons^11,12^, and sub-100 nm resolution using laser-plasma SXR sources^13–16^.

Our approach for an SXR XCT is the use of laser-plasma source based on a double stream gas puff target, in which two gasses are injected into the laser-matter interaction region^17^ to form a plasma emitting EUV and SXR radiation. The inner gas is the material of the target, to which a specific elemental emission can be attributed. The second gas surrounds the inner gas, to decrease the density gradient of the target gas in the direction of the nozzle axis. This significantly increases the target density in the interaction region and enhances EUV/SXR emission. Such laser-plasma EUV/SXR source was already employed for various applications, including metrology^18^, full-field EUV^19^ and SXR^16^ nanoimaging, photoionization^20^, polymer surface modification^21^, radiobiology^22^.

In this work, we present results of employing an SXR emission from krypton/helium plasma, to perform the XCT with ~2 nm axial spatial resolution of 10-nm period multilayer Mo/Si structure. The results are in very good agreement with numerical simulations. A compact, desktop in size XCT setup, was developed, with a footprint of 1.5 × 1.5 m^2^, including a driving laser system. The XCT setup allows achieving an axial resolution comparable to the illumination bandwidth. Such compact, desk-top XCT system provides the possibility to perform test experiments or to check initial and novel approaches to the XCT data processing with samples, which may be later studied in more detail at large scale facilities. On top of that, such compact desk-top system might, in the near future, allow for a broader spread of the XCT technique to environmental, material sciences and biology, especially in the “water-window” range. Thus, it will be possible to perform XCT imaging using a desktop system in the small company or in the university laboratory in order to obtain preliminary data on novel materials and samples, without the immediate need to get beam-time on the large-scale facility. Additionally, such compact system for XCT might allow researchers to do investigations on samples that may be too fragile or have other constraints and limitations that preclude measurements at a synchrotron source.

## Results

### The spatial and temporal coherence of the SXR radiation emitted from a laser-plasma source

**Figure 1:**
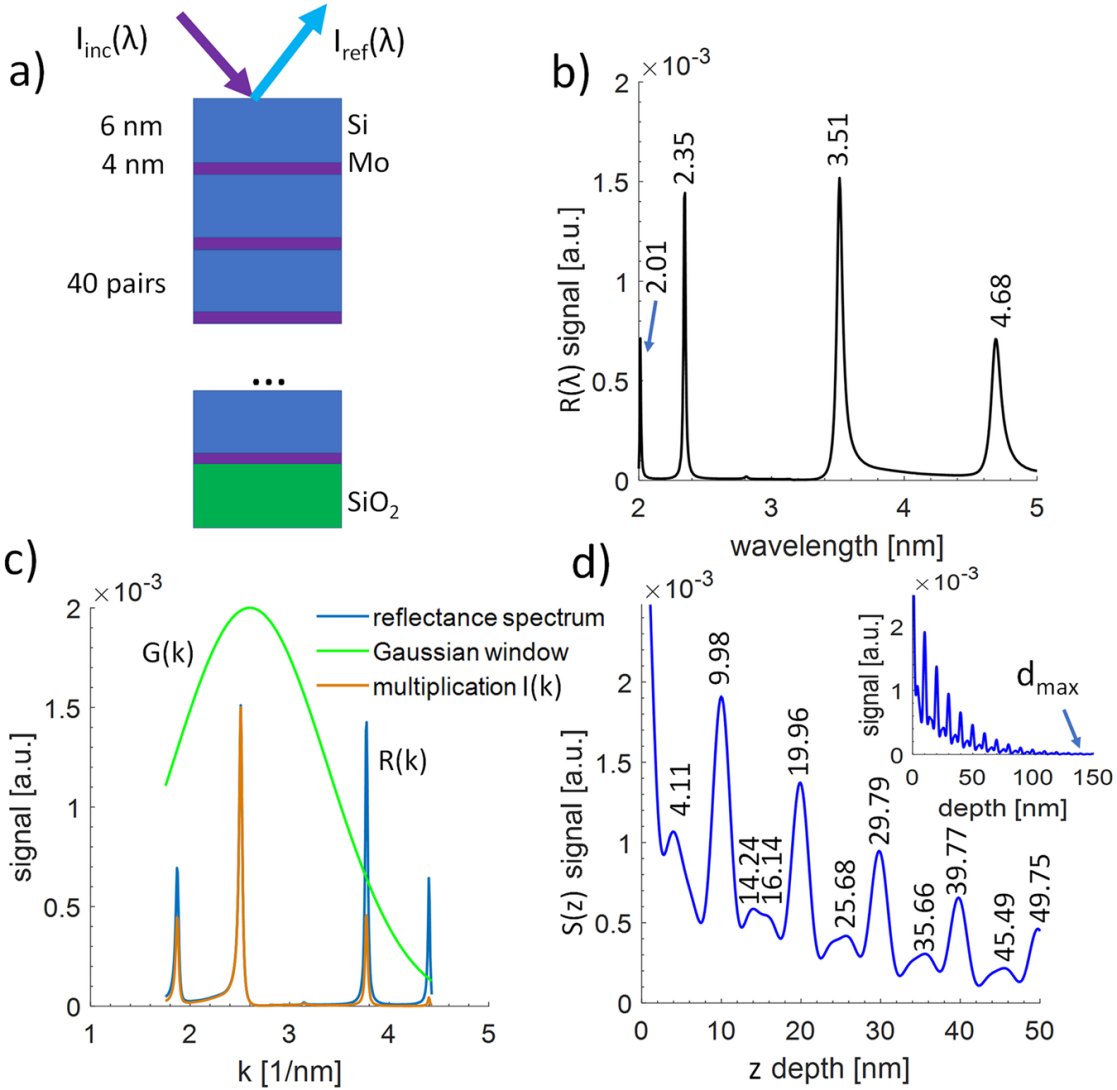
Schematic depiction of the Mo/Si multilayer structure, studied using the “water-window” XCT approach (**a**). A simulated reflectivity of such structure in the SXR wavelength range from 2 nm to 5 nm (**b**). Converted reflectivity spectrum into a *k*-space with a Gaussian-type window applied (**c**) and reconstruction of a depth information, related to the vac/Si/Mo/Si/Mo… interfaces in the investigated structure (**d**). The small inset in (**d**) shows the maximum depth *d*_*max*_ from which, according to simulation, the signal can be acquired.

In the XCT experiments, the sample under investigation is illuminated by a broadband X-ray radiation (incident spectral intensity) *I*_*inc*_*(λ)*, as depicted in Fig. 1(a). XCT operates on the principle of interference between wavefronts (beams), reflected from the internal structure of the investigated samples and from a reference beam, reflected from the top (first) discontinuity in the index of refraction. This allows for certain wavelengths in the broadband illumination to be enhanced due to constructive interference, *I*_*ref*_* (λ) –* reflected spectral intensity in Fig. 1(a), while the other wavelengths, due to destructive interference, are being attenuated. This produces a certain reflectivity spectrum, a fingerprint, which encodes the interferometric information about the internal structure of the investigated sample. For that, a coherent radiation is required. A spatial coherence of the SXR radiation from the laser plasma source was approximated by the limited size of the Kr plasma, 0.5 mm in diameter. Taking into account the wavelength range of the SXR emission from 2.2 nm to 5.5 nm and according to Equation (2)

2 $${D}_{c}=2{R}_{c}\approx \frac{\lambda \cdot z}{D},$$

where *D*_*c*_ is the diameter of coherence area, *z* is the distance between plasma and the sample, *D* is the diameter of the plasma and *λ* is the wavelength, the radius of coherence *R*_*c*_^23^ was estimated to be in the range of 0.6–1.5 μm, at the location of the sample.

Moreover, from the emission bandwidth of Δλ ~ 2 nm, the coherence length *l*_*c*_ from Eq. (1) in λ = 2–5 nm wavelength range, (see Kr plasma emission spectrum in Methods), was estimated to be *l*_*c*_ = 2–12.5 nm. Such temporal coherence allows having a constructive interference between closest layers, at the same time it is not too large to limit the cross-interference terms between layers spaced further apart.

### Numerical simulations of the XCT measurements

To check a feasibility of performing the XCT measurements in the “water-window” numerical simulations were performed. In this work, a flat Mo/Si multilayer mirror (Fraunhofer IOF), 20 × 20 mm^2^ in size was used as a sample. It is a standard Mo/Si mirror, for 13.5 nm wavelength with ~37% reflectivity^24^ for unpolarized EUV radiation with 1 nm spectral bandwidth. It is composed of 40 pairs of Si/Mo multilayers deposited on top of SiO_2_, each 10 nm thick, with 0.4 bottom layer thickness to period ratio, in a similar structure depicted schematically in Fig. 1(a)^25^. A calculated reflectivity spectrum of such mirror is shown in Fig. 1(b), where four spectral peaks are present. It was computed based on the transfer-matrix formalism for the calculation of optical response in multilayer structures^26^ and double-checked with CXRO data^24^. The peak with the highest reflectivity of ~1.5·10^−3^ is at λ = 3.51 nm. Such low reflectivity is understandable since the multilayer structure of the mirror was design to reflect efficiently 13.5 ± 0.5 nm radiation. We used this sample, however, because the small period of the Mo/Si, only 10 nm (6 nm of Si and 4 nm of Mo), is comparable to the illumination wavelength and quite challenging to faithfully reconstruct and demonstrate a few nanometers axial resolution.

The reflectivity spectrum was next converted into a *k*-space, which takes into account the dispersion and the refraction of SXR radiation in the sample materials, according to Equation (3). The conversion depends on the incidence angle α, measured to the surface normal, and wavelength-dependent index of refraction of the dominant material *n*_*D*_, which is silicon in our case. The index of refraction was computed based on atomic scattering factors^24,27^. Since the refractive index is wavelength-dependent, the optical paths between layers in the sample and, of course, the reconstructed distances between the layers differ for the various wavelengths of a broadband incident radiation. Thus, the dispersion has to be taken into account. Fortunately, in the EUV and SXR region the indices of refraction of various materials do not differ much, thus, it is sufficient to consider only the index of diffraction of the dominant material^8^ to achieve adequate and reliable depth profile.

3 $$k=\frac{4\pi }{\lambda }\sqrt{{n}_{D}^{2}-si{n}^{2}\alpha }$$

The result of *I*(*λ*) → *I*(*k*) conversion operation is shown in Fig. 1(c) as “reflectance spectrum” *R(k)*. Next, a Gaussian-type window *G(k)* was applied to shape the reflection spectrum, to produce *I*(*k*) = *R*(*k*) · *G*(*k*) before a Fourier reconstruction is performed. The windowing of the input data, producing “multiplication” curve *I(k)* in the Fig. 1(c), allows reducing high frequency artifacts in the Fourier axial reconstruction. The Gaussian window, Equation (4):

4 $$G(k)=\exp [-\frac{{(k-{k}_{0})}^{2}}{{\sigma }^{2}}]$$

has parameters of *k*_0_ = 2.6 nm^−1^ and *σ* = 1.1 nm^−1^.

The depth reconstruction was performed through an inverse Fourier Transform using Equation (5):

5 $$S(z)={ {\mathcal F} }^{(-1)}\{I(k)\},$$

where *S(z)* is the depth profile and *z* is the depth. Moreover, to improve the final reconstruction, a *k*-space equalization and 4× Fourier domain upsampling were performed. The reconstruction of the depth profile is depicted in Fig. 1(d). Each peak in the curve represents the depth, at which the discontinuity of index of refraction occurs, producing a reflected beam, which theoretically interferes with the reference beam reflected from the first surface. Most of the peaks are located at proper positions, namely 10, 16, 20, 26 nm, etc. in the progression corresponding to the real internal structure of the designed multilayer, however, the first peak is located at 4.11 nm instead of 6 nm depth. Moreover, there are some artifacts in the reconstruction, such as an artificial peak at 14.24 nm. Such peak might be explained by the fact, that the constructive interference in the structure is also possible not only between reflected beam from each discontinuity and from the first layer but also cross-interference between layers might produce additional (ghost) peaks in the depth reconstruction. In our case, the sample has a periodic structure, which helps to minimize the influence of cross-interference on the depth profile reconstruction.

The depth reconstruction is scaled Fourier Transform of reflectivity spectrum in the *k*-space, so the reconstruction depends on its structure. The spectrum was simulated only in the spectral range from 2 to 5 nm wavelength, where only four spectral peaks are present. Such narrow range was chosen on purpose, because the SXR emission form Kr/He plasma source starts from 2 nm wavelength, while the longest detectable wavelength by the grazing incidence spectrometer (GIS) is ~5.5 nm. Due to a limited number of spectral contributions in the reflectivity spectrum, the depth reconstruction has some artifacts, mentioned above. Still, however, shows reliably the internal structure of the multilayer Mo/Si sample. Due to absorption of SXR radiation in the multilayer structure, the signal from deeper parts of the sample is weaker than from the near surface region. This can be seen in the simulation since it takes into account a complex refraction index, which imaginary part is responsible for the absorption. The small inset in Fig. 1(d) shows the maximum depth *d*_*max*_ ~ 120–150 nm, from which, according to simulation, the signal can still be acquired.

### XCT experiment using the “water-window” SXR radiation

To verify the simulation, XCT experiment was carried out using the setup described in Methods. The reflectivity of the Mo/Si sample was measured using GIS spectrometer. Due to a very low reflectivity of the sample in the SXR spectral range, 16 spectral images were recorded, each with an exposure of 500 SXR pulses, 8000 SXR pulses in total, at 10 Hz source repetition rate. To improve the signal to noise ratio by a factor of 4, all images were added up. A long entrance slit of the GIS spectrometer, approximately 5–6 mm in length, allows us to integrate spectrum by summing up 300 CCD detector lines. To obtain the reflectivity of the sample a Kr emission spectrum was taken into account, as described by Equation (6)

6 $$R(\lambda )={I}_{ref}(\lambda )/{I}_{inc}(\lambda ),$$

where *I*_*inc*_(*λ*) and *I*_*ref*_ (*λ*) are incident and reflected spectral intensity, as depicted in Fig. 1(a).

**Figure 2:**
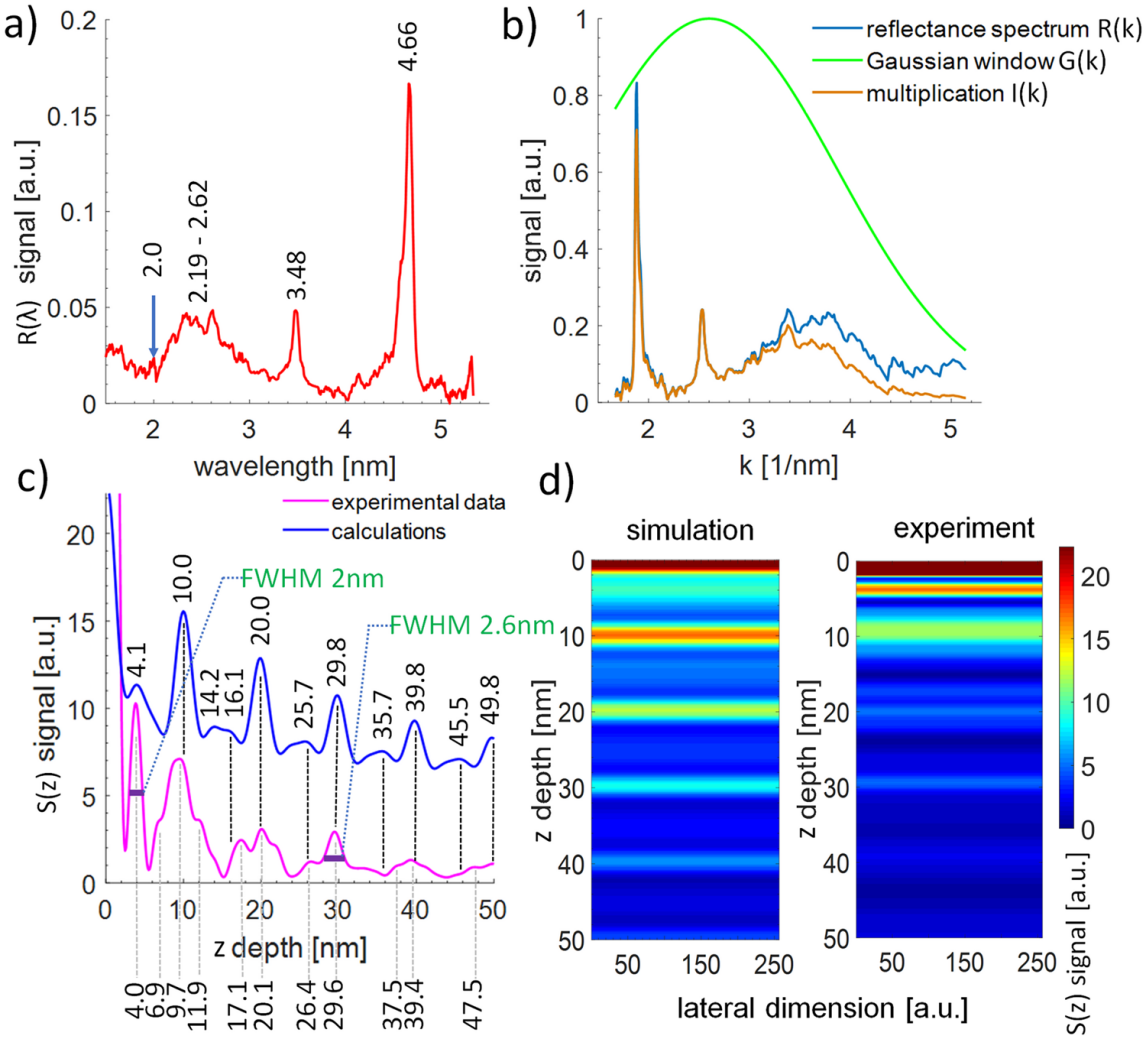
A measured reflectivity of the Mo/Si multilayer structure in the SXR wavelength range from 1.5 nm to 5.5 nm (**a**). A *k*-space reflectivity spectrum with a Gaussian-type window applied (**b**), similarly to data shown in Fig. 1 (**c**). A reconstruction of a depth information from the Mo/Si structure, experimental data (bottom, pink curve) and simulation (top, blue curve). Visualization of depth structure of the sample (**d**); a comparison between theoretical and experimental data.

A measured reflectivity of the Mo/Si multilayer structure in the SXR wavelength range from 1.5 nm to 5.5 nm is presented in Fig. 2(a). The measured spectral peaks are located close to the simulated ones, depicted in Fig. 1(b), except one at λ = 2.35 nm, which in the measurement - Fig. 2(a), shows up as a spectral band λ = 2.19–2.62 nm.

It is due to the low signal amplitude since the detection of the spectral features from 2–2.6 nm required sub-noise detection techniques, thus such heavy integration and long exposure was necessary to register this spectral contribution.

The experimental reflectivity converted into *k*-space and windowing Gaussian function are presented in Fig. 2(b). In the experiment, the Gaussian window function *G(k)* parameters are *k*_0_ = 2.6 nm^−1^ and *σ* = 1.8 nm^−1^. A reconstruction of a depth information from the Mo/Si structure, based on experimental data (bottom, pink curve) compared to simulation (top, blue curve) are depicted in Fig. 2(c). The numbers, above and below the curves, indicate the horizontal position (depth values) of each peak for comparison between theoretical and experimental data. A visualization of a depth structure of the sample, depicted in Fig. 2(d), shows a comparison between theoretical and experimental data. As can be seen in Fig. 2(c,d) the experimental depth profile is in good agreement with numerical calculations. The experimental Fourier peaks were detected up to a depth of 90–100 nm, roughly 70% of the depth predicted by numerical simulations. Main peaks, spaced every period of the multilayer structure, are clearly matched in both datasets. The positions of the measured peaks in between the main peaks, for depths larger than 10 nm, correspond well to the numerical data, however, a shift of ~1–2 nm is present. Even the first peak, corresponding to the first Si-Mo interface, which was simulated to be at the depth of 4.11 nm is located at 4 nm depth in the measured data. This confirms that the simulation and reconstruction approaches are correct, however, in the λ = 2–5.5 nm wavelength range there is not enough spectral information to faithfully reconstruct a proper location of the first Si-Mo interface.

From the experimental data, an axial spatial resolution can be estimated. The FWHM of the peak, related to the first Si-Mo interface equals to ~2 nm. Taking into account another, well defined experimental spectral peak at 30 nm depth, the FWHM is estimated to be ~2.6 nm. The axial spatial resolution in XCT can be comparable to FWHM values and is of the order of 2–2.6 nm. However, the position of the peaks can be found with a much better accuracy of less than 1 nm. Thus, the two layers cannot be resolved if they are closer than 2–2.6 nm, however, if spread more apart, their absolute position in respect to the top layer (or interface) can be found with a much higher resolution, better than 1 nm.

### XCT reconstruction – a possible improvement

An improvement to the experimental reconstruction can be made by expanding the wavelength range from λ = 2–5.5 nm to λ = 1–7 nm. This is experimentally not possible in our system, since the limited wavelength range of our GIS spectrometer, however, the simulation, which we will present in the current section, demonstrate this as one possible approach to further improve the depth profile reconstruction.

**Figure 3:**
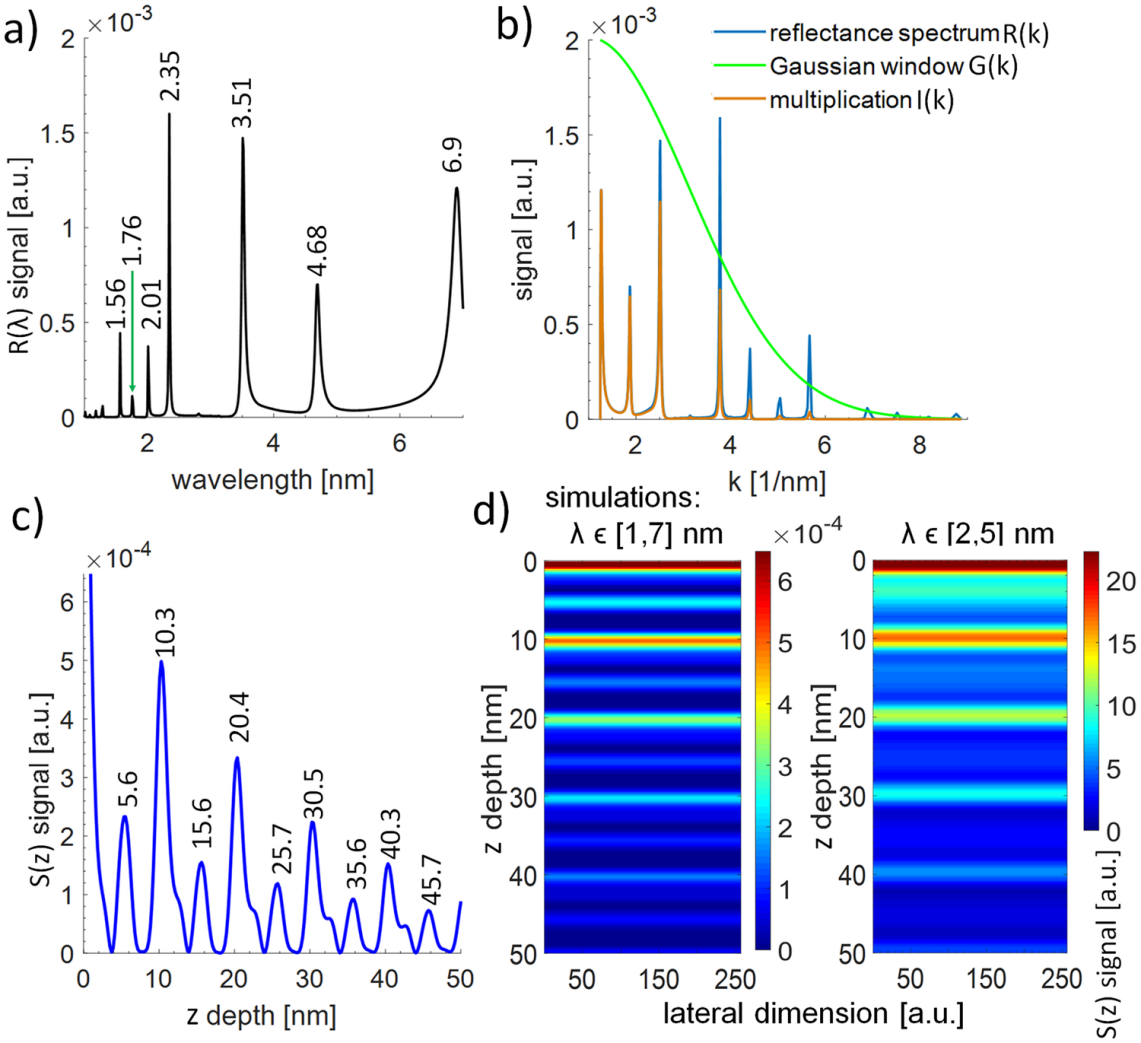
A simulated reflectivity of the Mo/Si multilayer structure in slightly wider SXR wavelength range from 1 nm to 7 nm (**a**). A *k*-space reflectivity spectrum with a Gaussian-type window applied (**b**), enhancing the peaks at low *k*-values, below 2 nm^−1^ (spectral peaks at 4.68 nm and 6.9 nm wavelength). XCT reconstruction from the Mo/Si structure, based on numerical reflectivity spectrum in the enhanced spectral range (λ = 1–7 nm) (**c**). Due to additional peaks in the reflectivity spectrum, outside of the experimental wavelength range (2–5.5 nm wavelength), the simulation show locations of the peaks with better accuracy, compared to Fig. 1(d). It is especially evident in the first peak, located at 4.11 nm in Fig. 1(d), which should be located around *z* = 6 nm (5.6 nm in **c**). Simulated depth structure of the sample (**d**) for two spectral ranges (λ = 1–7 nm and λ = 2–5 nm).

A simulated reflectivity spectrum in an expanded wavelength range from λ = 1–7 nm is depicted in Fig. 3(a). Except for previously indicated spectral components, there are additional ones, especially at λ = 6.9 nm and a few smaller ones for λ < 2 nm, i.e. λ = 1.76 nm and λ = 1.56 nm. Those, however, are much weaker and contribute much less to the Fourier reconstruction than the longer wavelength spectral component. As before, the simulated reflectivity converted into *k*-space and windowed by a Gaussian function *G(k)*, with parameters *k*_0_ = 1 nm^−1^ and *σ* = 3 nm^−1^, are presented in Fig. 3(b). As one can notice, the window function was shifted more towards smaller values of *k*, putting more weight on the longer wavelength components, especially the one at λ = 6.9 nm, which was not taken into account in the previous simulations and experimental data.

A reconstruction of a depth information from the same Mo/Si structure, but imaged in the extended wavelength range is depicted in Fig. 3(c). The positions of the main peaks (every 10 nm), as well as less visible inner peaks (6 + *n*·10) nm, where *n* ∈ (0, 1, 2…39}, are in agreement with the simulated multilayer structure. Even though the main peaks are asymmetric, the peaks clearly indicate the multilayer’s interfaces positions. Figure 3(d) shows a comparison between simulated data for both narrower wavelength range λ = 2–5 nm, and an expanded beyond the “water-window” spectral range of λ = 1–7 nm.

## Discussion and Conclusions

In conclusion, a proof of principle experiment, showing the possibility to perform XCT in the “water-window” spectral region using a compact laser plasma SXR source was demonstrated with an axial resolution of ~2 nm. The XCT data was obtained using a broadband emission from a laser-produced Kr plasma obtained as a result of a laser-matter interaction with double stream gas puff target. The depth profile of a Mo/Si mirror, used as a sample, was successfully retrieved from the XCT experimental data, obtained in the wavelength range from 2 nm to 5.5 nm wavelength. The dept profile is in very good agreement with the numerical simulations. Moreover, an improvement of this experiment was also proposed, which considers slightly extended wavelength range from 1 nm to 7 nm SXR wavelengths, beyond the “water-window” range. This extended range provides more reliable depth reconstruction, matching better the real sample geometry and removing some of additional, false peaks in the Fourier reconstruction. This is possible by taking into account more spectral peaks in the reflectivity spectrum, thus more spectral information produces better-matched Fourier reconstruction. It shows, that it is possible and desirable to sometimes go beyond typical “silicon-” and “water-window” spectral range to collect sufficient spectral information to perform the high quality reconstruction.

In our experiment, Kr plasma emission was used because of efficient emission in the SXR “water-window” range with a significant bandwidth, having IRB close to one for the shortest wavelength of λ = 2 nm. This allowed one to perform the depth reconstruction with axial resolution approaching 2 nm, which is related to the widths of some Fourier peaks presented in Fig. 2(c). The widths and positions of most of the experimental peaks are also very similar to the simulated ones. Such agreement between the simulated and experimental data was possible even without taking any additional precautions to improve the reference signal. Typically, the top layer of the investigated structure is coated with high reflectivity material, such as gold, to enhance the reference signal for improved interference contrast and reconstruction profile. In our case, the sample was without any additional processing.

An important issue is, however, that one has to collect sufficient spectral information for a reliable reconstruction, otherwise, the depth reconstruction might have some errors. In our case, it is the axial position of the first Fourier peak at ~4 nm depth, instead of 6 nm as in the theoretical structure of the Mo/Si mirror.

Our initial XCT experiments show good axial resolution, but for now, the XCT signal gives averaged information about the sample in the plane perpendicular to the optical axis. The acquisition of the XCT data is not yet spatially localized. It is acquired from a large area, limited by de-magnified spectrometer entrance slit size, roughly 40 μm × 4 mm. In the near future, we plan to implement SXR focusing and perform the XCT by acquiring the data from relatively small sample area, which, in turn, will allow producing 3-D reconstructions.

## Methods

**Figure 4:**
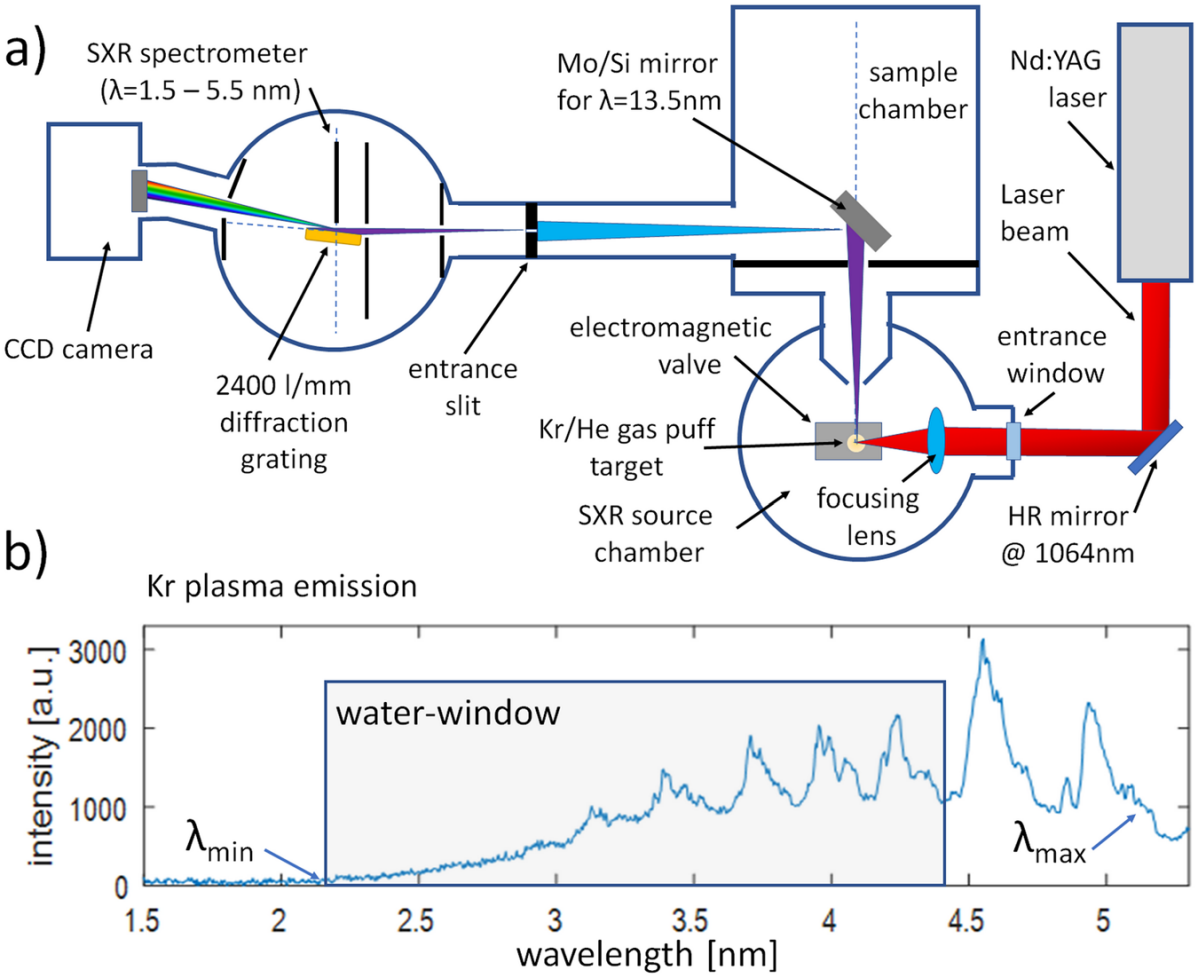
Optical scheme of the experimental arrangement for “water-window” XCT experiment (**a**) and Kr plasma emission from ~2 nm to 5 nm, encompassing the entire “water-window” spectral range (**b**).

The experimental XCT setup is depicted in Fig. 4(a). An Nd:YAG laser beam, emitted from NL 303 HT laser system (EKSPLA), with laser pulse energy of 0.65J and 3 ns time duration is focused by an f = 2.5 cm focal length lens onto a double stream gas puff target^17^. The target is formed by two collinear nozzles, driven independently by two electromagnetic valves, which open and close the flow of two gases. The inner, circular nozzle was supplied with krypton gas at an optimum backing pressure of 4 bar, while the outer nozzle was supplied with helium gas pressurized to 5.5 bars.

Due to the interaction of the laser pulses with the gaseous target a laser-produced plasma (LPP) is created, which emits radiation in the broad range of wavelengths, from SXRs to infrared, depending on the gas used as a target, laser beam and focusing system parameters. In our experiment, an efficient SXR emission from krypton was achieved in the spectral range from ~2.2 nm to 5.5 nm wavelength, as indicated in Fig. 4(b). More details about the source, its optimization for efficient SXR emission from Kr/He gas puff target and characterization can be found in^28^.

The radiation from krypton plasma enters the second vacuum chamber, in which a sample under investigation is placed 270 mm away from the plasma. The sample reflects the SXR radiation at 45° in respect to the sample surface, towards a home-made grazing incidence SXR spectrometer (GIS). The spectrometer is equipped with a grazing incidence diffraction grating, having 2400 lines/mm (Hitachi), an 80 µm entrance slit and a CCD camera, in configuration reported in^29^. The entrance slit of the spectrometer was located 300 mm from the sample surface. The arrangement of the SXR spectrometer is depicted in Fig. 4(a). The spectral range of the spectrometer was from 1 to 5.5 nm wavelength. The spectra produced by the SXR grating are recorded using a back-illuminated CCD camera (GE 20482048, greateyes GmBH), located downstream the diffraction grating. The camera was equipped with a chip with 2052 × 2046 pixels, each 13 × 13 μm^2^ in size. During the experiments the chip was cooled down to −40 °C to reduce its internal noise and the background. The resolving power of the SXR spectrometer, equipped with 80 μm slit in the vicinity of the carbon absorption edge was equal to E/ΔE ~120, measured as an FWHM width of an isolated line at 4.409 nm wavelength, 2s^2^2p^3^-2s^2^2p^2^(3 P)3d transition^30^ from S^9+^ ions from SF_6_ gas, photon energy of 281 eV.

### Data availability

The data that support the findings of this study are available from the corresponding author upon reasonable request.

## References

[1] Huang D (1991). Optical coherence tomography. Science.

[2] Drexler, W. & Fujimoto, J. G. *Optical Coherence Tomography* (Springer International Publishing Switzerland 2015).

[3] Keane P (2012). Evaluation of Age-related Macular Degeneration With Optical Coherence Tomography. Surv. Ophthalmol..

[4] Wojtkowski M (2009). Comparison of reflectivity maps and outer retinal topography in retinal disease by 3-D Fourier domain optical coherence tomography. Optics Express.

[5] Fuchs S (2012). Optical coherence tomography using broad-bandwidth XUV and sof X-ray radiation. Appl. Phys. B.

[6] Attwood, D. & Sakdinawat, A. *X-Rays and Extreme Ultraviolet Radiation* (Cambridge University Press, 2017).

[7] Jaeglé, P. *Coherent Sources of XUV Radiation, Soft X-Ray Lasers and High-Order Harmonic Generation* (Springer, New York, NY, 2006).

[8] Fuchs S (2016). Nanometer resolution optical coherence tomography using broad bandwidth XUV and soft x-ray radiation. Scientific Reports.

[9] Fuchs S (2017). Optical coherence tomography with nanoscale axial resolution using a laser-driven high-harmonic source. Optica.

[10] Feder R (1977). High-resolution soft x-ray microscopy. Science.

[11] Chao W, Harteneck BD, Liddle JA, Anderson EH, Attwood DT (2005). Soft X-ray microscopy at a spatial resolution better than 15 nm. Nature.

[12] Rehbein S, Heim S, Guttmann P, Werner S, Schneider G (2009). Ultrahigh-resolution soft-x-ray microscopy with zone plates in high orders of diffraction. Phys. Rev. Lett..

[13] Berglund M, Rymell L, Peuker M, Wilhein T, Hertz HM (2000). Compact water-window transmission X-ray microscopy. Journal of Microscopy.

[14] Takman PAC (2007). High-resolution compact X-ray microscopy. Journal of Microscopy.

[15] Legall H (2013). A compact Laboratory Transmission X-ray Microscope for the water window. Journal of Physics: Conference Series.

[16] Wachulak PW (2015). A Compact “water window” microscope with 60 nm spatial resolution for applications in biology and nanotechnology. Microsc. Microanal..

[17] Fiedorowicz H, Bartnik A, Jarocki R, Rakowski R, Szczurek M (2000). Enhanced X-ray emission in the 1-keV range from a laser-irradiated gas puff target produced using the double-nozzle setup. Appl. Phys. B.

[18] Fiedorowicz H (2005). Compact laser plasma EUV source based on a gas puff target for metrology applications. J. Alloys Compd..

[19] Wachulak PW, Bartnik A, Fiedorowicz H, Kostecki J (2011). A 50 nm spatial resolution EUV imaging–resolution dependence on object thickness and illumination bandwidth. Opt. Express.

[20] Bartnik A, Fiedorowicz H, Wachulak P (2014). Spectral investigations of photoionized plasmas induced in atomic and molecular gases using nanosecond extreme ultraviolet (EUV) pulses. Phys. Plasmas.

[21] Bartnik A (2012). Simultaneous treatment of polymer surface by EUV radiation and ionized nitrogen. Appl. Phys. A.

[22] Adjei D (2015). Development of a compact laser-produced plasma soft X-ray source for radiobiology experiments. Nucl. Instr. Meth. Phys. Res. B.

[23] Born, M. & Wolf, E. Principles of Optics (7th ed.) 576 (Cambridge University Press, Cambridge, 1999).

[24] The Center for X-ray Optics (CXRO), X-ray Interaction with Matter calculator, http://henke.lbl.gov/optical_constants/multi2.html.

[25] Bajt S (2002). Improved reflectance and stability of Mo-Si multilayers. Opt. Eng..

[26] Troparevsky C (2010). Transfer-matrix formalism for the calculation of optical response in multilayer systems: from coherent to incoherent interference. Optics Express.

[27] Henke BL, Gullikson EM, Davis JC (1993). X-ray interactions: photoabsorption, scattering, transmission, and reflection at E=50-30000 eV, Z = 1–92. At. Data Nucl. Data Tables.

[28] Wachulak, P. *et al*. A compact system for near edge X-ray fine structure (NEXAFS) spectroscopy using laser-plasma light source. Optics Express (in print) (2017). 10.1364/OE.26.008260 29715795

[29] Nakano N, Kuroda H, Kita T, Harada T (1984). Development of a flat-field grazing incidence XUV spectrometer and its application in picosecond XUV spectroscopy. Appl. Opt..

[30] Kelly, R. L. Atomic and Ionic Spectrum Lines below 2000 Angstroms: Hydrogen through Krypton. *J. Phys. Chem. Ref. Data* **16**, suppl. 1 (1987).

